# Preoperative Testosterone Therapy Prior to Surgical Correction of Hypospadias: A Review of the Literature

**DOI:** 10.7759/cureus.677

**Published:** 2016-07-08

**Authors:** Arvind Krishnan, Sean Chagani, Austin J Rohl

**Affiliations:** 1 University of Central Florida College of Medicine

**Keywords:** urology, hypospadias, testosterone therapy, androgen therapy, microphallus, male urethra

## Abstract

Hypospadias is a congenital anomaly of the male urethra that causes significant morbidity in the pediatric male population. The mainstay of treatment is hypospadias repair surgery. However, given the common co-occurrence of microphallus in these patients, surgery remains difficult without adequate tissue for proper reconstruction of the urethra. The use of preoperative testosterone therapy, parenterally or topically, has been a topic of study for several years in urologic literature. This literature review aims to summarize the different studies that have been conducted to address whether preoperative testosterone therapy is beneficial, inconsequential, or detrimental to the surgical and cosmetic outcomes of hypospadias repair as well as to address the differences in routes of administration.

## Introduction and background

Hypospadias is a congenital anomaly wherein the male urethra is abnormally displaced ventrally, exiting the posterior aspect of the penis anywhere within the glans, shaft, scrotum, or even the perineum. Hypospadias is the most common congenital anomaly, with an incidence ranging from 0.3 to - 0.7% of live male births [1-2]. Although many studies have failed to show a correlation between race and hypospadias risk, a longitudinal review of patients in California found that infants born to nonwhite mothers were at significantly decreased risk of hypospadias than those born to Caucasian mothers [3]. In the United States, surveillance data demonstrates a doubling in the prevalence of hypospadias from 0.2 to 0.4% between the years 1970 and 1993 [4].

To this day, the mainstay of treatment for uncomplicated hypospadias is surgical correction to create a penis with the urethral opening as close to the ventral tip of the penis as possible, with anatomic aesthetics directed at creating a straightened penis (upon erection) that is similar in appearance to a normal circumcised penis [5]. The American Academy of Pediatrics recommends that corrective surgery for hypospadias be performed before 18 months of age [6]. The general consensus among pediatric urologists is to perform elective surgery on male genitalia at six to 12 months of age to minimize stranger anxiety, separation anxiety, and general psychiatric distress from undergoing the invasive procedure [6].

After correction of the penile curvature (chordee), which is usually done by performing a dorsal midline plication, a new urethra must be constructed from the existing urethral opening. There are numerous techniques described in the literature to correct both distal and proximal hypospadias. Overall, the most commonly performed procedures are the tubularized incised plate (TIP) urethroplasty [7], the onlay procedure, and the meatal advancement and glanduloplasty procedure (MAGPI). The TIP procedure consists of creating a new urethra by tubularizing the skin of the ventral fascia that would have formed the urethra if embryologic development had not been prematurely arrested and re-directing the existing urethra to the ventral tip of the glans [7]. The onlay procedure is used for more severe hypospadias. In this procedure, a vascular strip of inner foreskin is dissected away and patched onto the urethral plate and directed, again, to the ventral tip of the glans [8-9]. The MAGPI procedure involves advancement of the dorsal urethral wall with a vertical incision and horizontal closure. It is most commonly performed when the meatus is positioned at the coronal margin [10]. There is no consensus as to which of the many hypospadias repair procedures in the literature has the best cosmetic outcome, but for proximal hypospadias, the onlay procedure is typically preferred [11-12].

Due to the very young age of patients who undergo correction of hypospadias, as well as the common comorbidity of a microphallus with hypospadias, pediatric urologists often have a very small surgical site with which they can operate. As such, it has been hypothesized that increasing size and girth of the penis preoperatively may lead to a larger surgical site, thus, reducing intraoperative and postoperative complications of the hypospadias corrective surgery. Preoperative testosterone therapy, both parenteral and topical, has been shown through various studies to definitively increase penile length and circumference; however, the literature as to whether or not this increase in size affects postoperative complications is still under debate. The goal of this review is to incorporate various studies and clinical trials in order to determine if there is an overall consensus regarding the route of administration and effects of preoperative testosterone therapy prior to hypospadias surgery.

## Review

In order to determine whether preoperative testosterone therapy reduces surgical complications, studies sought to confirm that administration of testosterone leads to a significant increase in penile length and circumference when administered prior to corrective surgery. Nerli, et al. demonstrated that both parenteral and topical testosterone achieved their desired therapeutic effect when compared to pre-therapy size. One group of males (Group A) with microphallic hypospadias received daily topical testosterone at 2 mg/kg/week for three weeks, while Group B received testosterone enanthate intramuscularly at 2 mg/kg once a month for three months. This study demonstrated no statistical significance in the two routes of administration [13]. While a total of five of the 21 children (two with topical, three with injection) studied developed fine pubic hair, none of the study participants developed sexual precocity, no untoward bleeding during any of the surgeries, and no delay in the bone age of any of the children evaluated [13]. Results of this study demonstrated a mean length and glans circumference (in mm) increase from 20.40 (+/- 0.48) to 24.16 (+/- 0.45), and 27.49 (+/- 0.29) to 37.7 (+/- 0.27), respectively, for parenteral administration. For topical administration, an increase from 20.58 (+/- 0.45) to 24.34 (+/- 0.36) in penile length and 27.81 (+/- 0.24) to 37.8 (+/- 0.20) for mean glans circumference was recorded.

Thus, testosterone is known to increase penile size in prepubertal boys due to its androgenic effects. This has been shown to be beneficial in the surgical repair of hypospadias, as a larger phallus size makes correction easier and less risky [14]. Androgen stimulation allows for more tissue to operate with and more robust vascular structures to improve healing. Bastos, et al. indeed showed in a prospective randomized trial an absolute increase in the number of blood vessels and vascular volume density after the administration of 1% testosterone ointment [15]. This may be particularly beneficial in patients who have had prior unsuccessful penile surgeries or those with microphallus [16]. Additionally, several studies have implicated other endogenous hormones similar to testosterone, such as B-HCG, in the improvement in hypospadias repair due to its effect in penile enlargement [17]. However, the lack of appropriate treatment protocols and administration route optimization leaves the question as to whether these endogenous androgens are significantly beneficial in improving surgical repairs and their resultant complications. Furthermore, some disadvantages to the use of androgen therapy have been described, including the increased risk of operative bleeding due to its angiogenic effect and poor wound healing due to the negative effect of androgens re-epithelialization both in vivo and in vitro [18].

There have been several studies to determine the optimal route of administration of testosterone to achieve the highest benefit of penile enlargement with the least systemic effects of androgen therapy. A randomized study in 1982 by Monfort, et al. sought to determine if a four-week trial of daily usage of 5% dihydrotestosterone (DHT) cream would result in significant growth stimulation. They found that mean penile length and circumference were augmented by 50% compared to pretreatment measures [19]. Similarly, a study by Kaya, et al. inquired whether dihydrotestosterone topical cream had an effect on complication rates and cosmesis. The same hypospadias repair procedure, tubularized incised plate (TIP) urethroplasty, was performed on all patients regardless of the arm of the study they were in (DHT cream or no DHT cream). It was determined that the rate of complications, including fistulas, strictures, glanular dehiscence, urethral diverticula, and meatal stenosis, were lower in the group of patients treated with DHT preoperatively. Minimal systemic side effects were observed, aside from irritating symptoms on the penile skin [14].

Parenteral testosterone therapy has also shown similar benefits. A randomized study by Ahmad, et al. in 2010 of 23 patients with hypospadias determined that parenterally administered testosterone, given at four, three, and two weeks prior to surgery at 2 mg/kg, had a statistically significant effect on penile growth up to the day of operation. This was observed both in penile length and preputial diameter. Furthermore, this was found to have a profound improvement in texture, vascularity, and pliability of the penile skin during surgery. A mild side effect of pubic hair growth was observed during this study with no effect on bone age or serum testosterone levels [16]. Other studies looking at the effect of preoperative parenteral testosterone also showed a significant increase in penile growth and local vascularity [20-21].

Comparison studies have also been conducted. Chalapathi, et al. completed a study of 26 patients with hypospadias and microphallus who were randomly assigned to receive either topical application of testosterone or intramuscular injection of testosterone. While both groups received a combination of testosterone propionate and testosterone enanthate in a 2 mg/kg/wk dose for three weeks, penile growth in group A was achieved by a topical application while in group B it was achieved by intramuscular injection. Interestingly, it was found that both routes of administration achieved satisfactory penile enlargement, with a 60% increase in growth rate in topically treated patients and a 75% increase in growth rate in parenterally treated patients. However, the difference in effects on penile growth between the two routes of administration was not statistically significant. Of note, increased side effects were noted in the topically treated group, with abnormally high testosterone levels, increased pigmentation, and frank dermatitis. This was attributed to the unpredictable absorption of topical testosterone [22]. A study of 21 male patients with hypospadias conducted in 2009 found similar results when comparing parenteral versus topical therapy. They, too, found that both groups of patients had significant increases in penile length and circumference following testosterone administration over a three week period. However, as opposed to the previously mentioned study, they did not find any significant systemic effects of testosterone in either group [13].

Of note, many studies have explored factors that may predict success or failure after hypospadias surgery. A retrospective review by Chung, et al. analyzed 348 hypospadias repairs over the course of 20 years and determined that the location of hypospadias (proximal more than distal) predicts a higher rate of complications (urethrocutaneous fistulization) [23]. Similarly, Uygur, et al. found that in addition to location, the surgical technique used was also an important predictor of success. Flap procedures were found to be associated more commonly with complications. It was also found that surgical skill and expertise in choosing the right technique for each case were important factors [24].

However, only a few robust prospective clinical trials have looked at complication rates as they relate to preoperative hormonal stimulation. As mentioned previously, Kaya, et al. determined that complication rates in patients treated preoperatively with DHT therapy were significantly lower, but the effects of testosterone therapy were not determined in this study [14]. More recently, however, a prospective, randomized, controlled study was done by Asgari, et al. to address this topic [25]. The study included 182 children with hypospadias who were randomized into a testosterone group (n = 91), who received preoperative testosterone parenterally, and a control group (n = 91) who did not receive any preoperative treatment. All of the children were operated on by the same urologist using the TIP procedure with or without chordee correction. Postoperative complications included were urethrocutaneous fistulas, urethral diverticula, meatal stenosis, and glanular dehiscence. An increase in penile length and penile circumference were achieved in all but four patients in the testosterone group. The overall complication rate was significantly higher in the control group than in the testosterone group, with a rate of 13.18% versus 5.45% (P = 0.03). All of the children in the testosterone group experienced transient side effects, such as pigmentation of the genitalia and scant pubic hair. This study concluded that preoperative testosterone administration is beneficial in decreasing complication rates [25].

## Conclusions

In summary, there have been a number of studies looking at the use of preoperative testosterone therapy in male patients with hypospadias. From the aforementioned studies, there is a consensus that preoperative testosterone therapy is effective in achieving its primary goal of increasing penile length and circumference. More importantly, initial studies by Kaya, et al. and Asmagi, et al. regarding surgical outcomes have demonstrated that patients who undergo preoperative testosterone therapy prior to hypospadias repair experience fewer complications and improved cosmesis [14, 25]. More robust studies specifically targeting long-term complication rates and cosmetic outcomes would be beneficial to corroborate these findings.

However, no consensus has been determined as to which route of administration is safer (i.e., results in fewer systemic effects). Inconsistent data in the studies that compare parenteral versus topical therapy regarding the systemic effects of each have made it difficult to draw any concrete conclusions. Reports by multiple researchers have indicated that topical testosterone absorption is highly variable and, as such, over-absorption may lead to increased serum testosterone, abnormal hair growth, and hyperpigmentation of the genitalia. However, other studies failed to observe any difference in systemic effects between groups. Thus, the safest and most efficacious route of administration remains to be determined.

## References

[1] Baskin LS, Erol A, Li YW, Cunha GR (1998). Anatomical studies of hypospadias. J Urol.

[2] Baskin LS, Ebbers MB (2006). Hypospadias: anatomy, etiology, and technique. J Pediatr Surg.

[3] Porter MP, Faizan MK, Grady RW, Mueller BA (2005). Hypospadias in Washington State: maternal risk factors and prevalence trends. Pediatrics.

[4] Paulozzi LJ, Erickson JD, Jackson RJ (1997). Hypospadias trends in two US surveillance systems. Pediatrics.

[5] Shukla AR, Patel RP, Canning DA (2004). Hypospadias. Urol Clin North Am.

[6] American Academy of Pediatrics Section on Urology (1996). Timing of elective surgery on the genitalia of male children with particular reference to the risks, benefits, and psychological effects of surgery and anesthesia. Pediatrics.

[7] Cheng EY, Vemulapalli SN, Kropp BP, Pope JC 4th, Furness PD 3rd, Kaplan WE, Smith DP (2002). Snodgrass hypospadias repair with vascularized dartos flap: the perfect repair for virgin cases of hypospadias?. J Urol.

[8] Rushton HG, Belman AB (1998). The split prepuce in situ onlay hypospadias repair. J Urol.

[9] Baskin LS, Duckett JW, Ueoka K, Seibold J, Snyder HM 3rd (1994). Changing concepts of hypospadias curvature lead to more onlay island flap procedures. J Urol.

[10] Duckett JW, Snyder HM 3rd (1991). The MAGPI hypospadias repair in 1111 patients. Ann Surg.

[11] Ghali Ghali, AM AM (1999). Hypospadias repair by skin flaps: a comparison of onlay preputial island flaps with either Mathieu's meatal-based or Duckett's tubularized preputial flaps. BJU Int.

[12] Baskin LS, Ebbers MB (2006). Hypospadias: anatomy, etiology, and technique. J Pediatr Surg.

[13] Nerli RB, Koura A, Prabha V, Reddy M (2009). Comparison of topical versus parenteral testosterone in children with microphallic hypospadias. Pediatr Surg Int.

[14] Kaya C, Bektic J, Radmayr C, Schwentner C, Bartsch G, Oswald J (2008). The efficacy of dihydrotestosterone transdermal gel before primary hypospadias surgery: a prospective, controlled, randomized study. J Urol.

[15] Bastos AN, Oliveira LR, Ferrarez CE, de Figueiredo AA, Favorito LA, Bastos Netto JM (2011). Structural study of prepuce in hypospadias--does topical treatment with testosterone produce alterations in prepuce vascularization?. J Urol.

[16] Ahmad R, Chana RS, Ali SM, Khan S (2011). Role of parenteral testosterone in hypospadias: A study from a teaching hospital in India. Urol Ann.

[17] Koff SA, Jayanthi VR (1999). Preoperative treatment with human chorionic gonadotropin in infancy decreases the severity of proximal hypospadias and chordee. J Urol.

[18] Gilliver SC, Ruckshanthi JP, Hardman MJ, Zeef LA, Ashcroft GS (2009). 5alpha-dihydrotestosterone (DHT) retards wound closure by inhibiting re-epithelialization. J Pathol.

[19] Monfort G, Lucas C (1982). Dehydrotestosterone penile stimulation in hypospadias surgery. Eur Urol.

[20] Luo CC, Lin JN, Chiu CH, Lo FS (2003). Use of parenteral testosterone prior to hypospadias surgery. Pediatr Surg Int.

[21] Gearhart JP, Jeffs RD (1987). The use of parenteral testosterone therapy in genital reconstructive surgery. J Urol.

[22] Chalapathi G, Rao KL, Chowdhary SK, Narasimhan KL, Samujh R, Mahajan JK (2003). Testosterone therapy in microphallic hypospadias: topical or parenteral?. J Pediatr Surg.

[23] Chung JW, Choi SH, Kim BS, Chung SK (2012). Risk factors for the development of urethrocutaneous fistula after hypospadias repair: a retrospective study. Korean J Urol.

[24] Uygur MC, Unal D, Tan MO, Germiyanoğlu C, Erol D (2002). Factors affecting outcome of one-stage anterior hypospadias repair: analysis of 422 cases. Pediatr Surg Int.

[25] Asgari SA, Safarinejad MR, Poorreza F, Asl AS, Ghanaie MM, Shahab E (2015). The effect of parenteral testosterone administration prior to hypospadias surgery: A prospective, randomized and controlled study. J Pediatr Urol.

